# A synaptic nidogen: Developmental regulation and role of nidogen-2 at the neuromuscular junction

**DOI:** 10.1186/1749-8104-3-24

**Published:** 2008-09-25

**Authors:** Michael A Fox, Matthew SP Ho, Neil Smyth, Joshua R Sanes

**Affiliations:** 1 Department of Molecular and Cellular Biology and Center for Brain Science, Harvard University, Cambridge, MA 02138, USA; 2 Department of Anatomy and Neurobiology, Virginia Commonwealth University Medical Campus, Richmond, VA 23298-0709, USA; 3 Center for Biochemistry and Center for Molecular Medicine, Medical Faculty, University of Cologne, D-50924, Cologne, Germany; 4 School of Biological Sciences, University of Southampton, Southampton, SO16 7PX, UK

## Abstract

**Background:**

The skeletal neuromuscular junction is a useful model for elucidating mechanisms that regulate synaptogenesis. Developmentally important intercellular interactions at the neuromuscular junction are mediated by the synaptic portion of a basal lamina that completely ensheaths each muscle fiber. Basal laminas in general are composed of four main types of glycosylated proteins: laminins, collagens IV, heparan sulfate proteoglycans and nidogens (entactins). The portion of the muscle fiber basal lamina that passes between the motor nerve terminal and postsynaptic membrane has been shown to bear distinct isoforms of the first three of these. For laminins and collagens IV, the proteins are deposited by the muscle; a synaptic proteoglycan, z-agrin, is deposited by the nerve. In each case, the synaptic isoform plays key roles in organizing the neuromuscular junction. Here, we analyze the fourth family, composed of nidogen-1 and -2.

**Results:**

In adult muscle, nidogen-1 is present throughout muscle fiber basal lamina, while nidogen-2 is concentrated at synapses. Nidogen-2 is initially present throughout muscle basal lamina, but is lost from extrasynaptic regions during the first three postnatal weeks. Neuromuscular junctions in mutant mice lacking nidogen-2 appear normal at birth, but become topologically abnormal as they mature. Synaptic laminins, collagens IV and heparan sulfate proteoglycans persist in the absence of nidogen-2, suggesting the phenotype is not secondary to a general defect in the integrity of synaptic basal lamina. Further genetic studies suggest that synaptic localization of each of the four families of synaptic basal lamina components is independent of the other three.

**Conclusion:**

All four core components of the basal lamina have synaptically enriched isoforms. Together, they form a highly specialized synaptic cleft material. Individually, they play distinct roles in the formation, maturation and maintenance of the neuromuscular junction.

## Background

The formation, maturation and maintenance of chemical synapses require multiple interactions between pre- and postsynaptic elements. Many of these interactions are mediated by membrane- or matrix-associated proteins that occupy the narrow cleft separating the pre- and postsynaptic membranes [1-3]. At the skeletal neuromuscular junction (NMJ), where such interactions have been analyzed in detail, a basal lamina (BL) passing between the motor nerve terminal and the postsynaptic membrane comprises the cleft material of this synapse. As expected from this arrangement, several of the molecules required for the formation, maturation and maintenance of the NMJ are BL components [4,5].

Members of four families of proteins are present in BLs throughout the body: laminins, collagens IV, heparan sulfate proteoglycans (HSPGs), and nidogens/entactins (referred to here as nidogens) [6]. Synaptic and extrasynaptic portions of the muscle fiber BL are known to bear distinct isoforms of the first three of these [4,5]. Laminins are large heterotrimers composed of α,β, and γ subunits. The major laminin in extrasynaptic BL is the α2β1γ1 heterotrimer, called laminin 211. In contrast, synaptic BL is rich in β2 but poor in β1 laminins, and contains, along with α2, the α4 and α5 subunits, both present at low levels extrasynaptically. Thus, synaptic BL contains laminins 221, 421, and 521 [7-10]. Collagens IV are trimers assembled from a set of six α chains. All muscle BL contains the α1 and α2 chains, likely in an [α1(IV)]_2_[α2(IV)]_1 _trimer, whereas the α3–6 chains are selectively associated with synaptic BL, presumably in [α3(IV)][α4(IV)][α5(IV)] and [α5(IV)]_2_[α6(IV)]_1 _trimers [8,11,12]. The HSPG perlecan is present in both synaptic and extrasynaptic BL, whereas another HSPG, agrin, is concentrated in the synaptic BL [13-15]. Importantly, studies of targeted mutant mice have shown that synaptic isoforms of all three families act as muscle-derived (laminins and collagens IV) or nerve-derived (agrin) synaptic organizers *in vivo*. Laminin β2 promotes the maturation of motor nerve terminals [16,17], laminin α4 regulates the precise apposition of pre- and postsynaptic specializations [18], and together laminins α4 and α5 promote the maturation of postsynaptic specializations [19]. Synaptic collagen chains are required for nerve terminal maintenance [12], and agrin is a critical stabilizer of postsynaptic differentiation [20-25].

In contrast to this wealth of knowledge about laminins, collagens IV and HSPGs, little is known about localization or roles of nidogens at the neuromuscular synapse. In *Caenorhabditis elegans*, the lone nidogen gene is necessary for the formation of the NMJ, although the protein is not synaptically enriched [26]. Unlike invertebrate genomes, two nidogen genes are present in mammals, encoding nidogen-1 and nidogen-2 [27-30]. In many tissues, mammalian nidogens are colocalized in BLs [31-33], and genetic studies suggest that they play largely redundant roles. Few phenotypes have been observed in targeted mutants lacking either nidogen-1 or nidogen-2, whereas double mutants lacking both nidogens die perinatally with defects in lung, heart and limb development [32-36]. Moreover, expression of nidogen-2 is dramatically increased in nidogen-1 mutants [32], supporting the notion that nidogens are capable of compensating for each other. In contrast, we show here that nidogen-2 is selectively associated with synaptic BL in muscle and is required for the maturation and maintenance of the adult NMJ. Synaptic laminins, collagens IV and heparan sulfate proteoglycans persist in the absence of nidogen-2, suggesting that its role extends beyond maintaining the integrity of the BL. Finally, we provide genetic evidence that synaptic localization of each of the four families of BL components is independent of the other three. Taken together with the work cited above, these results show that all four families of BL components have synapse-specific isoforms and synapse-specific functions.

## Results and discussion

### Selective association of nidogen-2 with neuromuscular synapses

**Figure 1:**
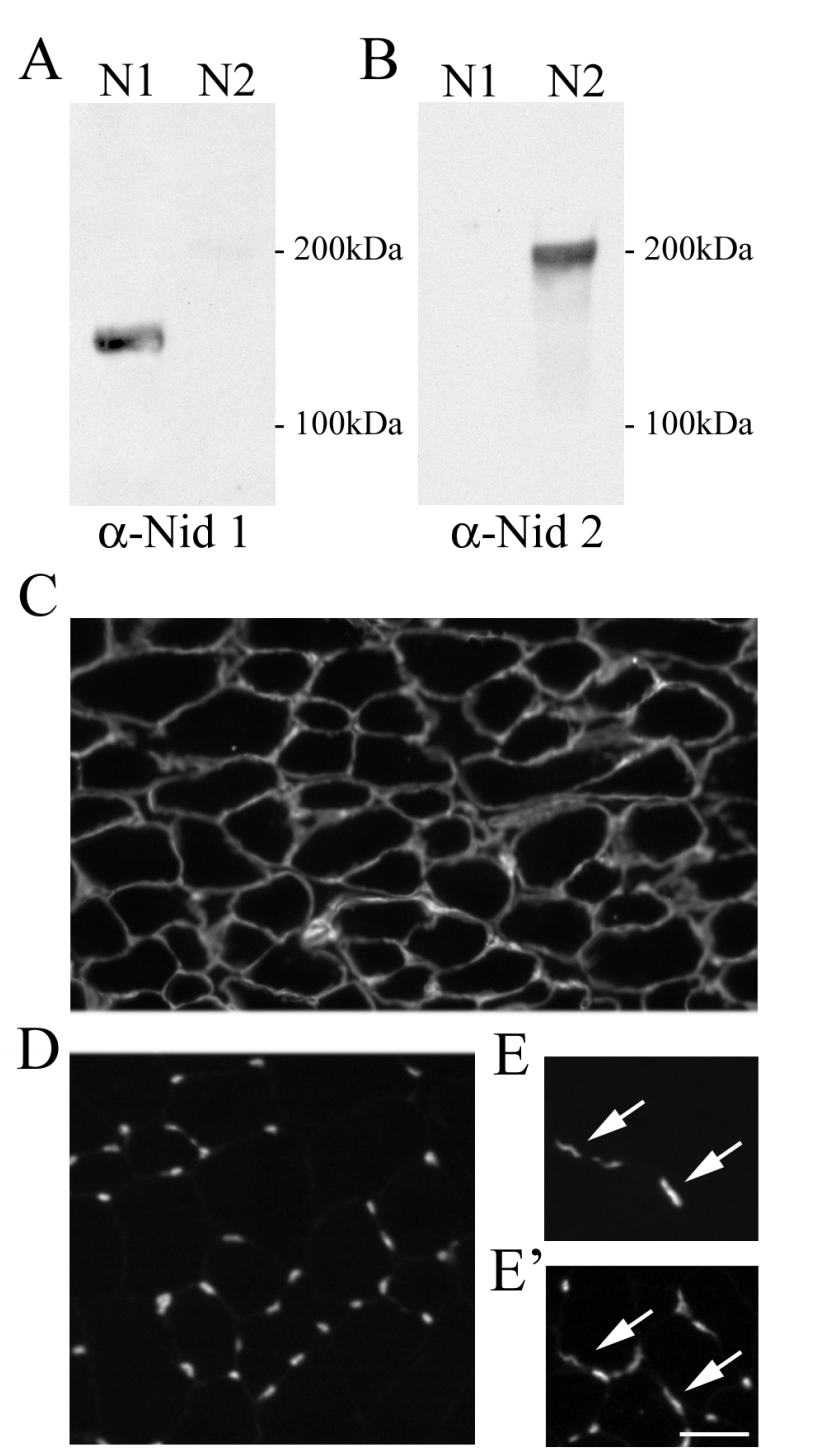
**Nidogen isoforms are differentially expressed in mouse muscle.** (A, B) Western blotting of recombinant nidogen-1 (N1) and -2 (N2) demonstrates specificity of nidogen-1 and nidogen-2 antibodies. (C, D) Young adult (postnatal day 56) mouse muscle cross-sectioned and immunostained with anti-nidogen-1 (C) or anti-nidogen-2 (D) antibodies. Nidogen-1 is present in the basal lamina surrounding muscle fibers. Nidogen-2 is largely absent from muscle fiber basal lamina, but is present in discrete basal laminas within muscle. (E, E') Double labeling of muscle with α-bungarotoxin (E) and anti-nidogen-2 (E') reveals that nidogen-2 is present at acetylcholine receptor-rich synaptic sites (arrows). Scale bar is 25 μm.

Nidogens-1 and -2 are similar in domain structure but differ in sequence (46% amino acid similarity in human, 43% in mouse [29,30,37]). To examine their distribution we sought isoform-specific antibodies. Equal quantities of full-length recombinant nidogen-1 and -2 were separated electrophoretically and immunoblotted with several commercially available antibodies. Antibodies were identified that reacted strongly and selectively with each nidogen isoform (Figure 1A, B). These antibodies were then used to localize nidogens in muscle.

As shown previously [32], nidogen-1 was associated with surfaces of adult muscle fibers whereas little if any nidogen-2 was detectable on most of the muscle fiber surface (Figure 1C, D). Nidogen-2 was, however, present on many small structures associated with muscle fibers. Most of these were capillaries that lie between muscle fibers (see below) but a minority resembled synaptic sites in size, shape and position. We therefore double-labelled muscle sections with nidogen antibodies plus a fluorescent derivative of α-bungarotoxin, which binds specifically to the acetylcholine receptors (AChRs) concentrated in the postsynaptic membrane at the NMJ. This demonstrated that synaptic sites did indeed contain nidogen-2 (Figure 1E).

**Figure 2:**
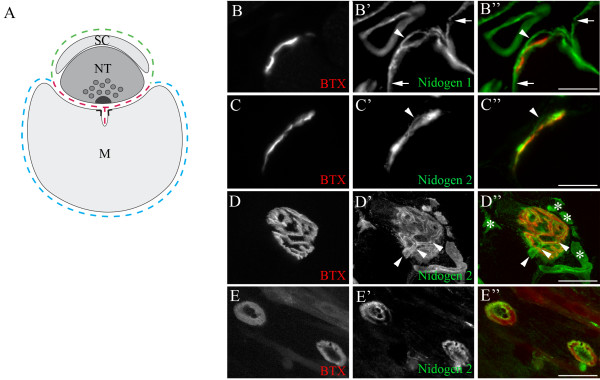
**Differential distribution of nidogens at the neuromuscular junction (NMJ).** (A) Schematic representation of the three cellular components of the NMJ (motor nerve terminal (NT), peri-synaptic Schwann cell process (SC), and skeletal muscle fibers (M)) and the basal laminas (BLs) that coat them (dashed lines). Dashed lines: red, synaptic BL; blue, extrasynaptic BL; green, Schwann cell BL. (B-C") Young adult NMJ cross-sections costained with α-bungarotoxin (BTX) and either anti-nidogen-1 (B) or anti-nidogen-2 (C). Nidogen-1 is present in synaptic, extrasynaptic (arrows in B', B") and Schwann cell BLs (arrowheads in B', B"). Nidogen-2 is absent extrasynaptically, but is enriched in synaptic and Schwann cell BLs (arrowheads in C', C"). (D-D") Confocal, *en face *image of an NMJ double-stained with BTX and anti-nidogen-2. Regions of co-localization demonstrate nidogen-2-rich synaptic BL. Tube-like structures overlaying synaptic sites (arrowheads) represent Schwann cell BL. Nidogen-2-positive structures near synaptic sites are capillaries (asterisks). (E-E") Nidogen-2 localized to acetylcholine receptor-rich (BTX-stained) sites in C2C12 myotubes. Scale bars are 5 μm in (B, C) and 20 μm in (D, E).

To localize nidogen-2 within the NMJ, we examined cross- and *en face*-sectioned NMJs, allowing us to distinguish three distinct domains in BL near synaptic sites: synaptic BL, which occupies the synaptic cleft; extrasynaptic BL, in directly adjoining stretches of the muscle fiber surface; and Schwann cell BL, atop Schwann cell processes that themselves cap nerve terminals (Figure 2A). Nidogen-1 was present in all three of these domains, whereas nidogen-2 was present in both synaptic and Schwann cell BLs but absent from extrasynaptic BL (Figures 2B–D).

On the basis of these findings, we investigated the relationship between nidogen-2 and the antigen recognized by the monoclonal antibody, 9H6, described by Chiu and Ko [38]. 9H6 selectively stains NMJs in rat and binds a carbohydrate-dependent epitope on a nidogen-1-like antigen. This antibody was described prior to the discovery of nidogen-2, so it seemed possible that it recognized nidogen-2. Because 9H6 fails to cross-react with mouse tissue, we stained rat muscle with anti-nidogen-2. In rats, as in mice, nidogen-2 was present at NMJs and in capillaries (data not shown), whereas 9H6 stained only NMJs [38]. Other differences in staining pattern are discussed below. Moreover, on immunoblots, 9H6 recognizes a protein with a molecular weight of 150 kDa [38], which is the expected size of mammalian nidogen-1 and considerably smaller than nidogen-2 (Figure 1A, B). These differences argue that the 9H6-antigen is not nidogen-2. Thus, synaptic BL may contain both a synapse-specific isoform of nidogen (nidogen-2) and a unique, glycosylated form of nidogen-1.

Synaptic BL can be deposited by muscles fibers, motor nerve terminals or Schwann cells [4,5,39]. Both muscle and Schwann cells have been reported to express nidogen-2 [40,41]. To determine whether muscle cells can contribute nidogen-2 to the synaptic cleft, we used a muscle cell line, C2C12. These cells were fused into myotubes on laminin substrata to promote AChR clustering [42]. Nidogen-2 was enriched at these AChR-rich sites even though no non-muscle cells were present in the cultures (Figure 2E). Thus, muscle cells are capable of nidogen-2 synthesis, secretion, and accumulation at synaptic sites.

As noted in the introduction, synaptic isoforms have been demonstrated for three of the families of core BL components – laminins, collagens IV and HSPGs [4,5]. Our results add the fourth, nidogen, to this list. The presence of synapse-specific isoforms of common BL components provides a molecular explanation for the observations that synaptic and extrasynaptic BLs are structurally similar and physically continuous but functionally distinct.

### Differential distribution of nidogens-1 and -2 in peripheral nerve

**Figure 3:**
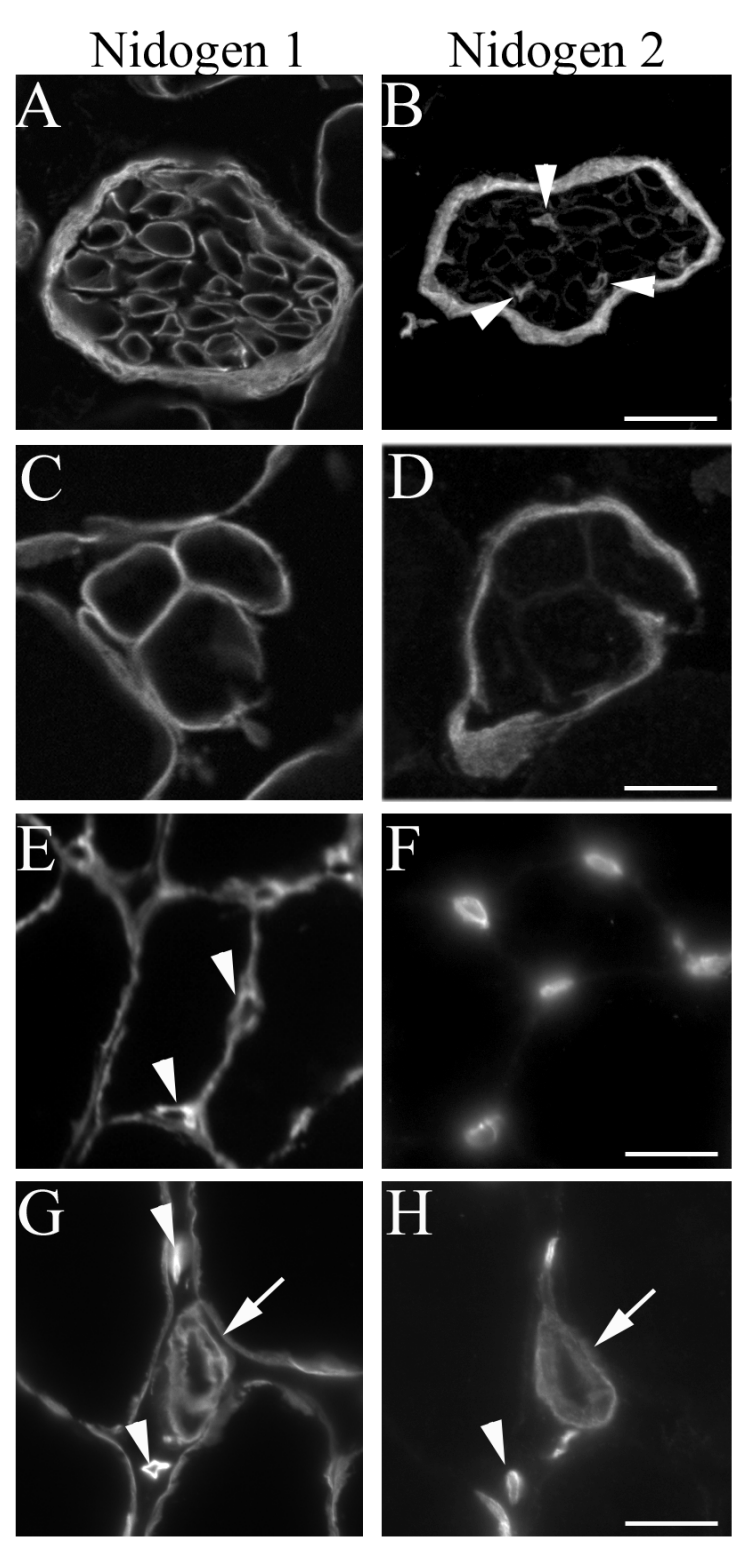
**Differential localization of nidogen isoforms in peripheral nerve.** (A, B) Nidogens are differentially localized in the basal laminas (BLs) associated with intramuscular nerve fascicles. Whereas nidogen-1 is present at similar levels in both peri- and endoneurial BL, nidogen-2 is enriched in perineurial BL. Most endoneurial BL expressed little nidogen-2, but a few structures within the nerve fascicle did contain nidogen-2 (arrowheads). Double staining with antibodies to CD31/PECAM indicate that most nidogen-2-positive structures within nerve fascicles are not associated with capillaries (data not shown). (C, D) Nidogens are differentially localized in BLs associated with sensory muscle spindles. Nidogen-1 is enriched in BLs surrounding individual intrafusal fibers within the muscle spindle, but nidogen-2 is enriched in the capsular BL surrounding the entire spindle. (E-H) Nidogen-1 and 2 are both present in BLs associated with vascular structures, including capillaries (E, F; arrowheads in E, G, H highlight capillary BL) and arterioles (arrows in G, H). Scale bar in (B) is 20 μm for (A, B), in (D) is 10 μm for (C, D), in (F) is 20 μm for (E, F), and in (H) is 20 μm for (G, H).

Next, we assessed the distribution of nidogen-1 and -2 in the BLs of peripheral nerves. The BL of the perineurium, which surrounds entire axon fascicles, was rich in both nidogens-1 and -2, whereas the BL of the endoneurium, which surrounds individual non-myelinating or myelinating Schwann cells, was rich in nidogen-1 but poor in nidogen-2 (Figure 3A, B). In contrast, antibody 9H6 labels endoneurial but not perineurial BL in rat [38], consistent with the idea that it recognizes a subset of nidogen-1 molecules but not nidogen-2.

A few structures within the nerve fascicle were rich in nidogen-2 (arrowheads in Figure 3B). Although some of these may be capillaries, most were not associated with CD31/PECAM-1, an endothelial cell marker ([43] and data not shown). Instead, they are likely to be the BL surrounding non-myelinating Schwann cells and their associated bundle of small-caliber, non-myelinated axons [41]. This localization is consistent with the observation that nidogen-2 is present in the BL of terminal Schwann cells (Figure 2C), which are non-myelinating.

Nidogen-1 and -2 were also differentially distributed in BLs of muscle spindles, sensory organs that respond to muscle stretch. Each spindle contains several specialized intrafusal muscle fibers, all of which are surrounded by a capsule BL. Intrafusal fiber BL was rich in nidogen-1, but poor in nidogen-2, whereas spindle capsule BL was rich in nidogen-2 but poor in nidogen-1 (Figure 3C, D). In contrast, BLs of intramuscular capillaries, venules, and arterioles all contained both nidogen proteins (Figure 3E–H and data not shown). To our knowledge, the spindle capsule is the only BL found to date that bears nidogen-2 but not nidogen-1.

### Developmental regulation of nidogens at the synaptic BL

**Figure 4:**
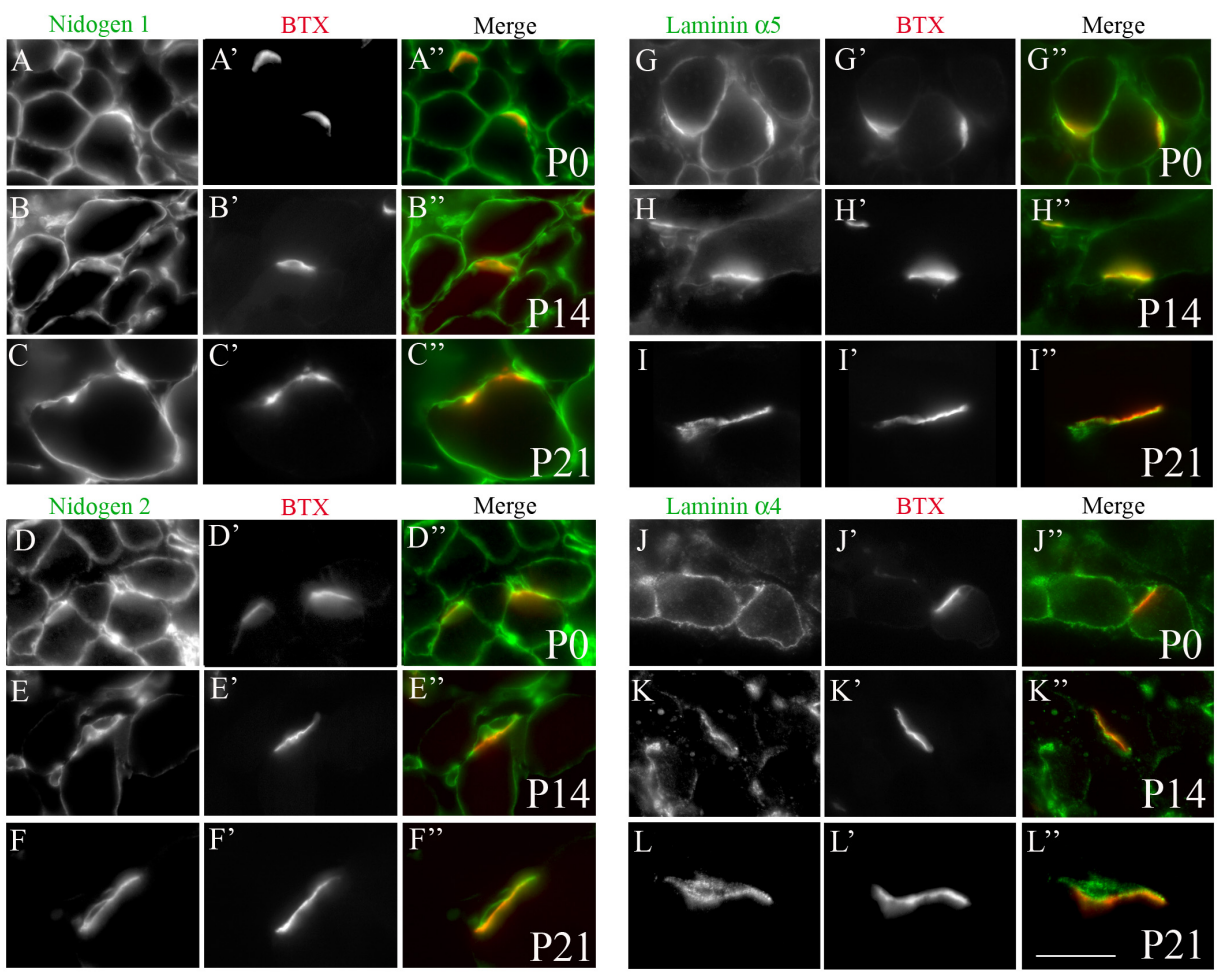
**Developmental regulation of nidogens and laminin α chains in synaptic basal lamina (BL).** Cross-sections of muscle from P0 (A, D, G, J), P14 (B, E, H, K) and P21 (C, F, I, L) mice, stained for α-bungarotoxin (BTX) and nidogens or laminins. (A-F) While nidogen-1 expression changes little during development (A-C), nidogen-2 is present in both synaptic and extrasynaptic BLs at birth (D) and becomes restricted to synaptic BL postnatally (E, F). (G-L) Laminins α4 (G-I) and α5 (J-L) become restricted to synaptic BL in parallel with nidogen-2. Scale bar is 15 μm for (A, D, G, J) and 10 μm for all other panels.

The distribution of some laminins, collagens IV, and HSPGs in muscle fiber BL changes as development proceeds [9,11,12,44]. We asked whether the same was true of nidogens. At birth, both nidogen-1 and nidogen-2 were present throughout both synaptic and extrasynaptic muscle fiber BL (Figure 4A, D). Similar patterns were present through the first postnatal week (data not shown). Moreover, levels of nidogen-1 in synaptic and extrasynaptic BL remained similar into adulthood (Figures 4B, C). In contrast, levels of nidogen-2 decreased in extrasynaptic BL during the second postnatal week, so synaptic and Schwann cell BL contained more nidogen-2 than extrasynaptic BL by postnatal day (P) 14 (Figure 4E). By P21, nidogen-2 was undetectable in extrasynaptic BL (Figure 4F). Thus, nidogen-2 becomes restricted to synaptic sites as the NMJ matures. Because our methods are not quantitative, we do not know whether the decreased abundance of nidogen-2 in extrasynaptic BL is accompanied by an increased abundance in synaptic and Schwann cell BLs.

We asked whether the time course with which nidogen-2 became restricted to the NMJ was similar to that of laminins α4 and α5, which are present extrasynaptically at birth but restricted to synaptic BL in adults [9]. In fact, the α4 and α5 laminin chains, like nidogen-2, were present throughout muscle fiber BL in neonates, were present at markedly higher levels synaptically than extrasynaptically by P14, and were largely synapse-specific by P21 (Figure 4G–L). Agrin is lost from extrasynaptic BL with a similar time course [44]. Thus, there may be a coordinated alteration in the composition of muscle fiber BL as development proceeds, similar to, but later than, the initial broad distribution and eventual synaptic concentration of AChRs [39]. In contrast, laminin β2 and synaptic collagen IV chains are synaptically concentrated from their initial appearance during embryogenesis and during the third postnatal week, respectively [9,12]. Thus, whereas the maturation of extrasynaptic BL may occur in a concerted fashion, the distinctions between synaptic and extrasynaptic BLs arise in a series of multiple steps.

### Nidogen-2 is necessary for maturation and maintenance of the NMJ

**Figure 5:**
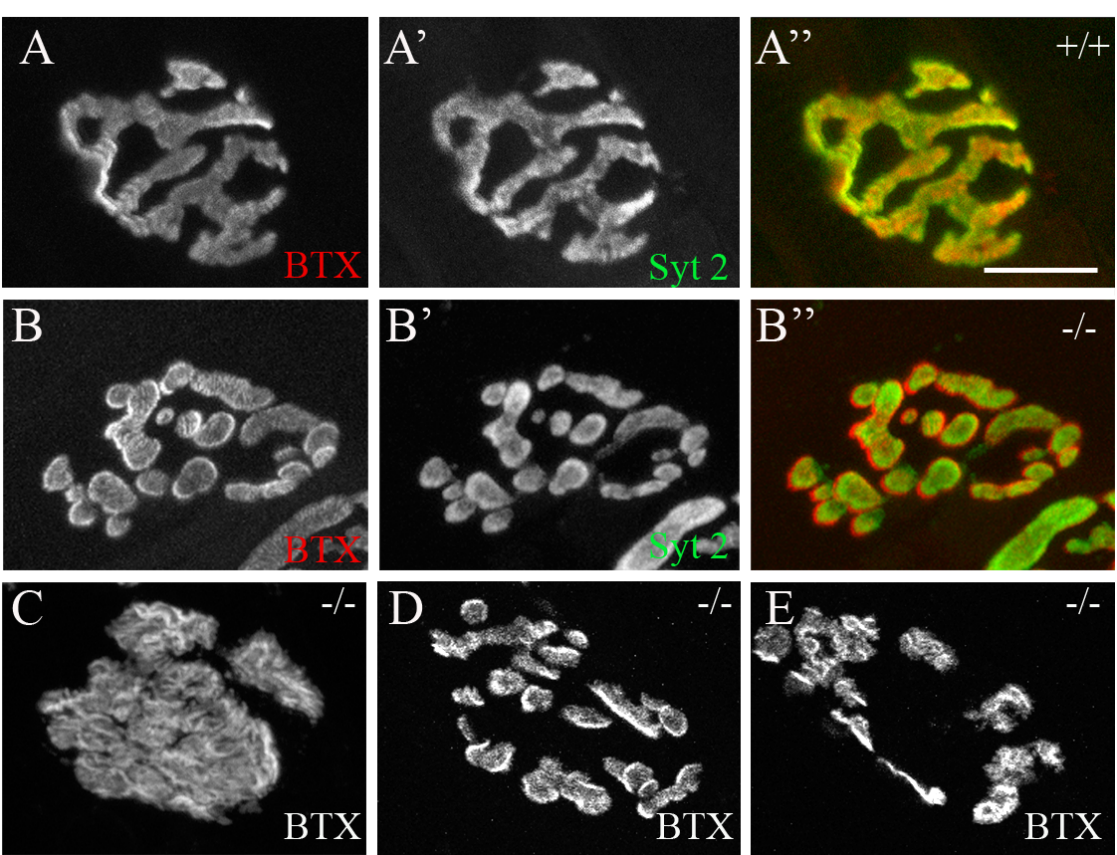
**Aberrant neuromuscular junction (NMJ) morphology in the absence of nidogen-2.** NMJs from diaphragms of P56 *nid2*^-/- ^mutants and aged-matched controls. Pre- and postsynaptic elements are labeled with anti-synaptotagmin 2 (syt 2) and α-bungarotoxin (BTX). (A) In controls, NMJs appeared pretzel-like. (B-E) In mutants, NMJs were frequently fragmented into small clusters (B, D, E) or appeared plaque-like (C). Despite topological abnormalities, mutant pre- and postsynaptic elements remain precisely aligned (B"). Scale bar in (A) is 10 μm for all parts.

Targeted nidogen-2 null mutant mice are viable and fertile, and no structural or functional defects have been detected in them to date [33,36]. To seek abnormalities in NMJs of nidogen-2 mutants, we labelled whole mounts of diaphragm muscles from young adults (P56) with markers of pre- and postsynaptic specializations, anti-synaptotagmin 2 (a synaptic vesicle protein) and α-bungarotoxin, respectively, then imaged NMJs by confocal microscopy. Although nerve terminals and postsynaptic membranes were closely apposed to each other, and obviously functional, the topology of the synapse was abnormal in mutants. In controls, NMJs appear 'pretzel-like', with continuous, branched AChR clusters (Figure 5A). In mutants, many NMJs appeared fragmented, with AChRs clustered into small, separate islands (Figure 5B, D, E). Others were plaque-like (Figure 5C), a shape characteristic of neonatal NMJs [45]. Thus, although nidogen-2-deficient NMJs are functional, their structure is aberrant.

**Figure 6:**
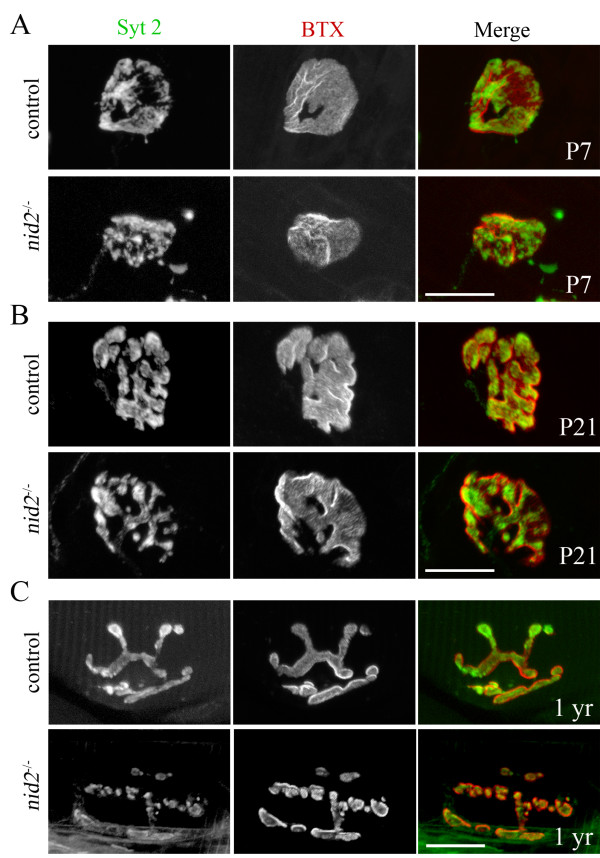
**Morphological defects in *nid2*^-/- ^neuromuscular junctions (NMJs) are due to improper maturation and maintenance.** (A, B) NMJs from diaphragms of P7 (A) and P21 (B) *nid2*^-/- ^mutants and aged-matched controls. Pre- and postsynaptic elements are labeled with anti-synaptotagmin 2 antibodies (syt 2) and α-bungarotoxin (BTX), respectively. No obvious defects were present at either age. (C) NMJs from diaphragms of 1-year-old mutants and age-matched controls. Mutant NMJs were fragmented at 1 year of age but were not appreciably more severely affected than in 2-month-old mutants (Figure 5). Scale bars are 20 μm.

Neuromuscular abnormalities observed in nidogen-2 mutants could reflect a role of nidogen-2 in the formation, maturation or maintenance of the NMJ. To distinguish these possibilities, we examined mutant muscles at P7, when NMJs are quite immature, and at P21, soon after the early postnatal period of synapse elimination is complete [39]. No obvious defects were observed in nidogen-2 mutants at either age (Figure 6A, B). Thus, nidogen-2 appears to be dispensable for formation and remodelling of the NMJ, but required for its full maturation or maintenance. To ask whether the defects are progressive, we examined diaphragms from 1-year-old nidogen-2 mutants. Defects were not appreciably more severe in 12-month-old mutants than in 2-month-old mutants. Thus, nidogen-2 is required for final stages in the maturation of the NMJ.

To test whether defects in NMJ morphology were secondary to muscle damage, we searched for muscle fiber degeneration in nidogen-2-deficient muscle. In healthy muscle, myonuclei are concentrated in the periphery of muscle fibers. In fibers that have undergone degeneration and regeneration, however, myonuclei are centrally located [46]. Less than 4% of mutant muscle fibers contained central nuclei (data not shown), whereas up to 80% of NMJs were abnormal (see below). Thus, defects in synaptic structure in nidogen-2-deficient mice were not the result of degenerating muscle fibers.

How might nidogen-2 promote maturation or enhance maintenance of the NMJ? Nidogens are capable of binding various cell surface receptors that are both present in pre- or postsynaptic membranes and necessary for synaptogenesis; these include integrins and leukocyte-common antigen related (LAR) receptor tyrosine phosphatase [3,37,39,47]. Therefore, one possible explanation for the defects in nidogen-2-deficient NMJs is that nidogen-2 exerts direct effects by binding receptors on either the motor nerve terminal or the postsynaptic apparatus. To test this idea, we used assays previously applied to identify and characterize other synaptic organizing molecules. For presynaptic differentiation, we applied soluble recombinant nidogen-1 or -2 to cultured chick motor neurons, and grew motoneurons on substrates coated with a mixture of recombinant nidogen and laminin-111. In both cases, we assayed the ability of nidogen to promote clustering of synaptic vesicles into aggregates such as those found in nerve terminals [48,49]. We also asked whether nidogens affected the length or branching of motor neurites. Neither nidogen-1 nor -2 detectably affected motor neurons under these conditions. For postsynaptic differentiation, we assayed the aggregation of AChRs in cultured myotubes [14,20,42]. In these assays, nidogen appeared to be detrimental to the health of the myotubes, so it was not possible to gauge their synaptic effects. Thus, these *in vitro *studies provide no evidence for a direct effect of nidogen on nerve or muscle, although we cannot draw definitive conclusions from them.

**Figure 7:**
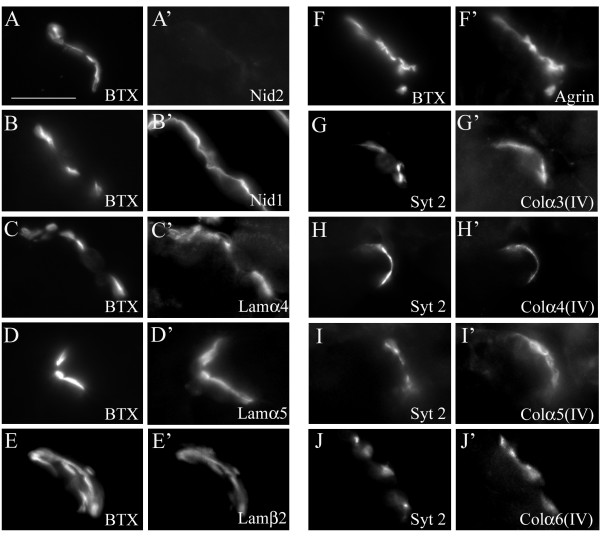
**Nidogen-2 is not required to restrict other basal lamina (BL) components to the synaptic BL.** (A-J) Synaptic sites in P56 *nid2*^-/- ^muscle cross sections were labeled with α-bungarotoxin (BTX) and antibodies to BL components: nidogen-1 (B) and -2 (A), synaptic laminin chains (Lamα4 [C], Lamα5 [D], and Lamβ2 [E] chains), agrin (F), and synaptic collagens IV (Colα3–6 [IV]) (G-J). No other components of the synaptic BL appeared altered in the absence of nidogen-2. Scale bar is 10 μm.

Another possible explanation for the defects in nidogen-2-deficient NMJs is that nidogen-2 might be required to recruit or retain other synaptic BL components that are, in turn, required for synaptic integrity. To test this possibility, we immunostained nidogen-2 mutant muscle with a panel of antibodies to synaptic BL components. As expected, no nidogen-2 was present in mutants (Figure 7A). Although nidogen-2 is upregulated in some tissues of nidogen-1 mutants [50], we failed to detect any changes of nidogen-1 level at nidogen-2-deficient synapses (Figure 7B, compare with Figure 2A). The major synapse-specific laminin subunits – laminin α4, α5, and β2 – as well as agrin and the synapse-specific collagens α3–6(IV) were all retained in synaptic BL in the absence of nidogen-2 (Figure 7C–J). Neither levels nor distribution of these proteins were detectably affected by the absence of nidogen-2, although we would not have detected small changes. From these results, we conclude that neuromuscular defects in nidogen-2 mutants are not indirect consequences of loss of synaptic laminins, collagens IV or agrin.

A third possibility is that nidogen-2 selectively binds and presents a matrix-associated synaptic organizing molecule to nerve or muscle. Indeed, several bioactive matrix molecules have been reported to bind with much higher affinity to nidogen-2 than nidogen-1, including tropoelastin, collagen XIII and collagen XVIII/endostatin [37,51-53]. Interestingly, we have found that mice with a targeted mutation of the collagen XIII gene have neuromuscular defects [54], and recent studies have shown that collagen XVIII is critical for motor axon growth and NMJ formation in *C. elegans *and zebrafish [26,55,56]. We therefore used immunohistochemical methods to ask whether synaptic localization of collagens XIII and XVIII are perturbed in *nid2*^-/- ^mice. Collagen XIII was concentrated at NMJs in control muscle, as reported previously [54,57] and this concentration persisted in the absence of nidogen-2, although we cannot rule out the possibility that its level may be modestly affected (data not shown). In both control and *nid2*^-/- ^muscles, collagen XVIII was undetectable in synaptic BL, although it was associated with the BL of terminal Schwann cells (data not shown). Thus, the mechanisms by which nidogen-2 contributes to synaptic maturation and maintenance remain to be determined.

### Intermuscular differences in the role of nidogen-2

**Figure 8:**
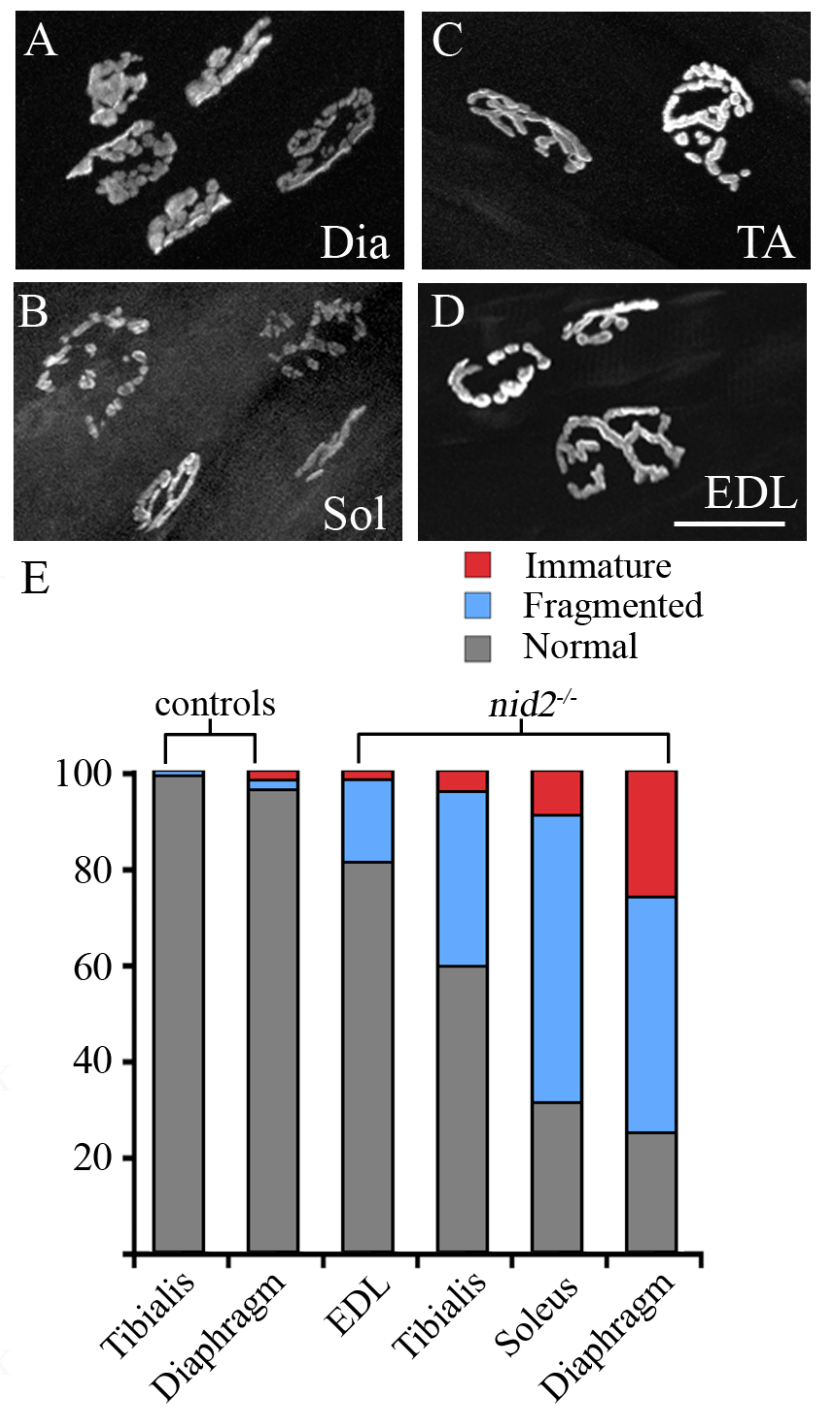
**Nidogen-2 phenotypes vary among muscles.** (A-C) Neuromuscular junction (NMJ) morphology was differentially affected in different muscles. Like diaphragm (Dia) (A), most NMJs in soleus (Sol) muscles were fragmented or immature (B), whereas NMJs in tibialis anterior (TA) and extensor digitorum longus (EDL) appeared less affected (C, D). NMJs were labeled with only α-bungarotoxin. (E) Quantification of NMJ morphology in several different mutant and control muscles. Y-axis represents the percentage of NMJs appearing either pretzel-like (that is, normal), fragmented (blue, as in Figure 6B, D, E), or immature (red, as in Figure 6C). Control tibialis anterior, n = 92 NMJs from 3 animals. Control diaphragm, n = 100 NMJs from 3 animals. *Nid2*^-/- ^EDL, n = 105, from 3 animals. *Nid2*^-/- ^tibialis anterior, n = 91, from 3 animals. *Nid2*^-/- ^soleus, n = 129, from 3 animals. *Nid2*^-/- ^diaphragm; n = 384, from 3 animals. Scale bar in (D) is 25 μm for (A-D).

Recently, we and others have observed striking intermuscular differences in neuromuscular phenotypes in mutants lacking agrin or collagen IV chains α3–6 [12,58,59]. The explanation for these differences remains unknown, but they are of interest because they may provide clues to mechanisms by which BL components act or muscles diversify. We therefore extended our study from diaphragm to three limb muscles – the extensor digitorum longus, soleus and tibialis anterior (Figure 8A–D). The soleus is a predominantly slow muscle; extensor digitorum longus and tibialis anterior are predominantly fast; and diaphragm is mixed. Nidogen-2 was concentrated at synaptic sites in limb muscles as well as in diaphragm (for example, Figure 2 shows limb muscle and Figure 4 shows diaphragm). Nonetheless, the percentage of abnormal junctions varied among muscles from approximately 20% in extensor digitorum longus to 75% in diaphragm (Figure 8E). Likewise, of those NMJs that were malformed, the ratio of those that were fragmented to those that were immature varied from 9:1 in extensor digitorum longus to 2:1 in diaphragm (Figure 8E). Intermuscular differences were less striking in 1-year-old mice, owing to a progressive accumulation of defects in mutant extensor digitorum longus and tibialis anterior muscles (data not shown). Thus, mutant muscles may vary in the rate at which defects accumulate rather than in their absolute susceptibility.

Muscles, and the NMJs within them, differ from each other in many respects [60] and it is not clear whether any documented factors explain the intermuscular differences we have observed. It is intriguing that NMJs more dependent on the presence of nidogen-2 are found in constantly active muscles (that is, diaphragm muscle controls respiration and soleus muscle controls lower limb posture), whereas fewer defects are observed in young, phasically active muscles (tibialis anterior and extensor digitorum longus). Another distinction is between two categories of muscles described by Pun *et al*. [58]. Called 'fast synapsing' or 'fasyn' and 'delayed synapsing' or 'desyn,' they were initially distinguished by the tempo and pattern of NMJ formation within them during embryogenesis. They were subsequently shown to differ in their sensitivity to nerve injury and neurological disease [58,61,62]. Interestingly, the two muscles we examined that were most severely affected, diaphragm and soleus, are both 'delayed synapsing' muscles, whereas the two muscles less affected in *nid2*^-/- ^mice, tibialis anterior and extensor digitorum longus, are 'fast synapsing' muscles [58].

### Independent localization of synaptic BL components

Biochemical studies have shown that interactions among three main structural components, laminins, collagens IV, and nidogens, are involved in BL assembly [63,64]. Moreover, all three of these components bind to HSPGs [4,63]. Our results have shown that specific isoforms of all four components are colocalized in synaptic BL. One might therefore presume that interactions among synaptic isoforms would mediate assembly of this specialized domain. Yet, we found that nidogen-2 is dispensable for the synaptic localization of synaptic laminins, collagens IV and agrin (Figure 7). We therefore considered the alternative possibility, that other synaptic BL components might be required for synaptic localization of nidogen-2. To test this idea, we examined the distribution of nidogen-2 in four mutants lacking synaptic laminin chains (β2, α4, α5, or both α4 and α5) and in collagen α5(IV) mutants, which lack all four synaptic collagen IV chains (α3–6) [12]. It was not possible to assess nidogen-2 in agrin mutants since these mice die at birth [21], prior to the synaptic restriction of nidogen-2.

**Figure 9:**
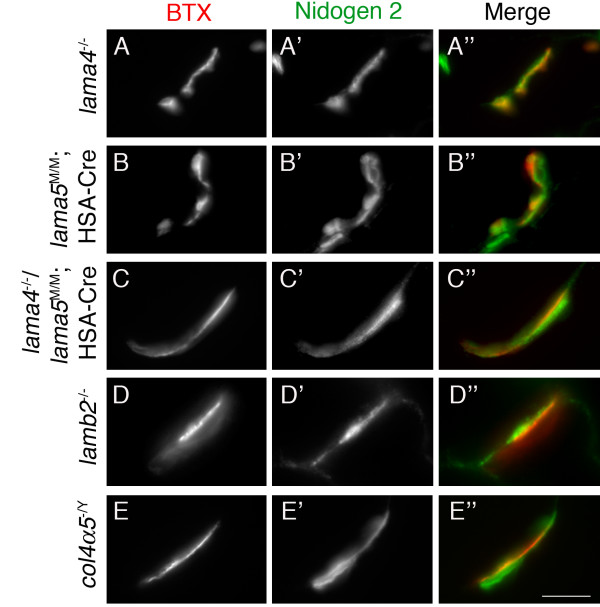
**Nidogen-2 localized to synaptic sites in the absence of other synaptic basal lamina components.** (A-E) Nidogen-2 was properly localized at synaptic sites (labeled with α-bungarotoxin (BTX)) in mutant mice lacking laminin α4 (*lama4*^-/-^) (A), laminin α5 (*lama5*^M/M^:HSA-Cre) (B), both laminin α4 and α5 (*lama4*^-/-^; *lama5*^M/M^:*HSA*-Cre) (C), laminin β2 (*lamb2*^-/-^) (D) and all four synaptic collagen IV chains (*col4a5*^-/Y^) (E). Scale bar is 5 μm.

Fragmentation and immaturity similar to that observed in nidogen-2 mutants has been reported in collagen α5(IV) and laminin α4 mutants and in laminin α4/α5 double mutants, respectively [12,18,19]. However, nidogen-2 remained concentrated in the synaptic BL of NMJs in all of these mutants (Figure 9). Thus, synaptic laminins and collagens IV are dispensable for concentrating nidogen-2 in the synaptic cleft. Moreover, the absence of nidogen-2 is not responsible for fragmentation of synapses lacking synaptic laminins or collagens IV.

**Figure 10:**
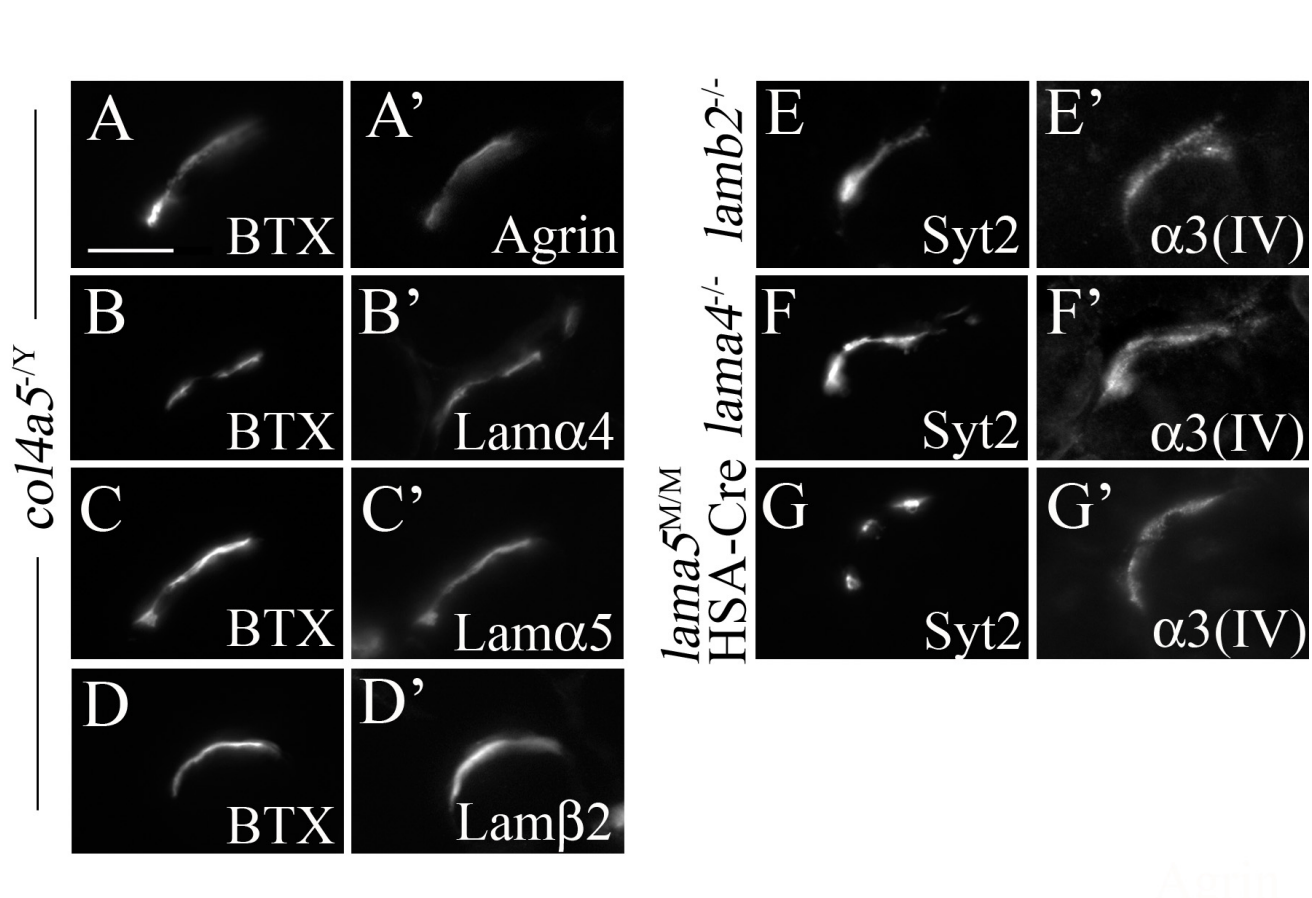
**Synaptic localization of laminins, collagens IV and agrin occurs independently of other major basal lamina (BL) components.** (A-G) In addition to nidogen-2 (Figure 9) other components of synaptic BL were examined in mutant mice lacking all four synaptic collagen IV chains (*col4a5*^-/Y^) (A-D) and collagen α3(IV) in laminin β2 (*lamb2*^-/-^) (E), laminin α4 (*lama4*^-/-^) (F) and laminin α5 (*lama5*^M/M^:*HAS*-Cre) (G) mutants. In the absence of any synaptic BL component, other families of BL molecules remained properly enriched at synaptic sites. Scale bar is 5 μm.

Finally, we broadened our inquiry to ask whether any synaptic BL components are necessary for the recruitment and restriction of other BL components to the synaptic cleft. As noted above, nidogen-2 is not required for synaptic localization of laminins, collagens IV or agrin, nor does it require synaptic laminins or collagens IV to become synaptically localized. On the other hand, previous results show that loss of a single laminin subunit or collagen IV chain can lead indirectly to absence of other components of the trimer from the synaptic cleft [9,12,65]. Here, we asked whether synaptic laminins are required for localization of synaptic collagens IV or visa versa. We found that synaptic collagens as well as agrin are normally localized to the NMJ in mutants lacking laminins β2, α4, or α5, and that synaptic laminins as well as agrin are normally localized to the NMJ in mutants lacking synaptic collagens α3–6(IV) (Figure 10 and data not shown). Together, these results, summarized in Table 1, indicate that the localization of each family of synaptic BL components occurs independently of other BL components.

**Table 1:** Enrichment of laminins, collagens IV, nidogen 2 and agrin in synaptic basal lamina of mutant mice

	Enrichment in synaptic basal lamina								
	
	Laminin			Collagen IV					
				
Mouse mutants	α4	α5	β2	α3	α4	α5	α6	Nidogen 2	Agrin
*lama4*^-/-^	**-**	**+**	**+**	**+**	**+**	**+**	**+**	**+**	**+**
*lama5*^M/M^*; *HSA-Cre	**+**	**-**	**+**	**+**	**+**	**+**	**+**	**+**	**+**
*lama4*^-/-^*; lama5*^M/M^*; *HSA-Cre	**-**	**-**	**+**	**+**	**+**	**+**	**+**	**+**	**+**
*lamb2*^-/-^	**+**	**-**	**-**	**+**	**+**	**+**	**+**	**+**	**+**
*col4a5*^-/Y^	**+**	**+**	**+**	**-**	**-**	**-**	**-**	**+**	**+**
*nid2*^-/-^	**+**	**+**	**+**	**+**	**+**	**+**	**+**	**-**	**+**

Includes data from this and previously published studies [9,12,16,18].

## Conclusion

We report here that nidogen-2 is selectively associated with synaptic BL at the NMJ and required for maturation or maintenance of this synapse. Combined with previous studies on laminins, collagens IV and HSPGs, these findings establish that each of the four major families of BL components has isoforms that are enriched in synaptic BL and required for synaptogenesis: laminins α4, α5, and β2, collagens α3–6(IV), agrin [7-9,11,12,15] (reviewed in [4,5,39], and nidogen-2. It remains unclear whether nidogen-2 interacts directly with receptors embedded in synaptic membranes or whether it acts by concentrating and presenting other synaptogenic factors. In *C. elegans *it has been proposed that nidogen exerts it synaptic organizing activity through a receptor protein tyrosine phosphatase present in presynaptic terminals [47]. We are currently testing the possibility that one or more members of this large family may be present at the NMJ. Importantly, synaptic functions of nidogen-2 cannot be fully compensated for by nidogen-1, so receptors or ligands mediating this effect are likely to selectively bind nidogen-2.

Our studies also provide the foundation for analyzing assembly of the synaptic cleft. Biochemical evidence has suggested that laminins, collagens IV, and nidogens are all necessary for BL assembly: laminins for initial BL formation; collagens IV for BL stabilization and maintenance; and nidogens for cross-linking laminin and collagen IV networks [36,66-68]. At the NMJ, however, the synaptic localization of each class of BL component appears to be independent of the others. Although it is possible that multiple, redundant interactions stabilize the synaptic cleft when a single component is removed, a more attractive idea is that synaptic laminins, collagens IV, HSPGs and nidogen are all localized by interactions with components of pre- or postsynaptic membranes. Previous studies have defined sites important for synaptic localization of laminin β2 and suggested that the receptor tyrosine kinase MuSK may be a localizing receptor for laminins or acetylcholinesterase [69-71]. Further analysis of how synaptic BL components are localized may provide a good model for understanding how the much less accessible synaptic cleft of central synapses is organized.

## Materials and methods

### Animals

Targeted mutant and transgenic mice used in this study have been described previously. They are: nidogen-2 mutants (*nid2*^-/-^) [31], laminin β2 mutants (*lamb2*^-/-^) [16], laminin α4 mutants (*lama4*^-/-^) [18], collagen α5(IV) mutants (*col4a5*^-/*Y*^) [72], obtained from Jackson Laboratories, Bar Harbor, ME, USA), conditional laminin α5 mutants (*lama5*^*flox*/*flox*^) [73], and mice that express Cre selectively in skeletal muscle (HSA-Cre) [74]. Mutants lacking laminin α5 selectively in skeletal muscle were generated by crossing *lama5*^*flox*/*flox *^and HAS-Cre mice; we refer to the *lama5*^*flox*/*flox*^;HSA-Cre mice as *lama5*^*M*/*M*^. Mice lacking both laminin α4 and α5 (*lama4*^-/-^*; lama5*^*M*/*M*^) were generated by crossing *lama4*^-/-^*;lama5*^*flox*/+^; HSA-Cre males and females. All mutants and transgenics were maintained on a C57/B6 background. In most cases, littermates of mutants were used as controls. CD1 mice were obtained from Charles River Laboratories, Inc. (Wilmington, MA, USA). All analyses conformed to NIH guidelines and were carried out under an animal protocol approved by the Harvard University Standing Committee on the Use of Animals in Research and Teaching.

### Antibodies

Primary antibodies used in this study are listed in Table 2. All fluorescently labeled secondary antibodies were obtained from Invitrogen/Molecular Probes and were used at a 1:1000 dilution. HRP-conjugated secondary antibodies from Vector Laboratories (Burlingame, CA, USA) were used at a dilution of 1:5000.

**Table 2:** Antibodies used in this study

Antigen	Isotype	Source	Dilution	Reference
Nidogen-1	Rabbit IgG	Abcam, Inc.	1:2000	Figure 1
Nidogen-2	Rabbit IgG	Abcam, Inc.	1:2000	Figure 1
znp-1 (Synaptotagmin 2)	Mouse IgG2a	Zebrafish International Resource Center	1:200	[76,77]
Collagen α3(IV)	Rat IgG	Gift of Y Sado (Shigei Medical Research Institute, Okayuma)	1:100	[78]
Collagen α4(IV)	Rat IgG	Gift of Y Sado	1:100	[78]
Collagen α5(IV)	Rabbit IgG	Generated in our lab	1: 2000	[11]
Collagen α6(IV)	Rat IgG	Gift of Y Sado	1:25	[78]
Laminin β2	Rabbit IgG	Gift of T Sasaki and R Timpl (Max Plank Institute, Munich)	1:1000	[79]
Laminin α4	Rabbit IgG	Kind gift of T Sasaki and R Timpl	1:1000	[80]
Laminin α5	Rabbit IgG	Generated in our lab	1:2000	[81]
Agrin	Rabbit IgG	Gift of Z Hall (UCSF)	1:300	[82]

### Western blotting

Full-length human nidogen-1 and -2 fusion proteins were purchased from R&D Systems (Minneapolis, MN, USA). Recombinant proteins (250 ng) were denatured by boiling in Laemmli buffer and separated by SDS-PAGE. Electrophoretically separated proteins were transferred to Immuno-Blot PVDF membrane (Bio-Rad, Hercules, CA, USA) in Tris-Glycine buffer (25 mM Tris, 190 mM glycine, pH 8.4)/20% methanol at 300 mA for 2 hours. Immunoblotted proteins were detected as previously described [75].

### Immunostaining

Mice were perfused with phosphate-buffered saline (PBS) and tibialis muscles were dissected. Tissue was immediately frozen in OCT on dry ice and 4 μm sections were cut on a cryostat. Sections were collected on gelatin-coated slides and allowed to air-dry for 15 minutes before tissue was fixed by incubating in ice-cold acetone for 10 minutes. For collagen IV antibodies, tissue was treated for 10 minutes in a 1:1 mix of 1 M KCl and 1 M HCl following fixation [12]. Sections were washed several times in PBS to remove any residual acid. After fixation, tissue was incubated with blocking buffer (5% non-fat milk in PBS with 0.2% Triton-X100 in PBS) for 30 minutes. Primary antibodies, diluted in blocking buffer, were incubated on the sections for 12 hours at 4°C, then sections were washed several times with PBS. Secondary antibodies, diluted in blocking buffer, were then incubated on the slides for 1 hour at room temperature. For controls, primary antibody incubation was omitted from the immunostaining protocol described above. Sections were washed thoroughly with PBS, cover-slipped with VectaShield, and visualized on an Olympus FV1000 scanning confocal microscope (Olympus America Inc., Melville, NY, USA).

### Cell culture

C2C12 cells (ATCC, Manassas, VA, USA) were cultured as previously described by Kummer *et al*. [42]. Myotubes were fixed with ice-cold acetone and stained as described above.

## Abbreviations

AchR: acetylcholine receptor; BL: basal lamina; HSPG: heparan sulfate proteoglycan; NMJ: neuromuscular junction; P: postnatal day; PBS: phosphate-buffered saline.

## Competing interests

The authors declare that they have no competing interests.

## Authors' contributions

JRS and MAF conceived the study, constructed the experimental design, and drafted the final manuscript together. MAF performed the experiments. MSPH and NRS provided nidogen mutant mice. All authors read and approved the final manuscript.
